# Oxygen changes drive non-uniform scaling in *Drosophila melanogaster* embryogenesis

**DOI:** 10.12688/f1000research.7221.1

**Published:** 2015-10-23

**Authors:** Steven G. Kuntz, Michael B. Eisen

**Affiliations:** 1 Department of Molecular and Cell Biology, University of California, Berkeley, Berkeley, CA, USA; 2 Howard Hughes Medical Institute, University of California, Berkeley, Berkeley, CA, USA; 3 Department of Integrative Biology, University of California, Berkeley, CA, USA

**Keywords:** oxygen, hypoxia, hyperoxia, embryo, development, time-lapse, temperature, Drosophila

## Abstract

We previously demonstrated that, while changes in temperature produce dramatic shifts in the time elapsed during *Drosophila melanogaster *embryogenesis, the relative timing of events within embryogenesis does not change. However, it was unclear if this uniform scaling is an intrinsic property of developing embryos, or if it is specific to thermal fluctuations. To investigate this, here we characterize the embryonic response to changes in oxygen concentration, which also impact developmental rate, using time-lapse imaging, and find it fundamentally different from the temperature response. Most notably, changes in oxygen levels drive developmental heterochrony, with the timing of several morphological processes showing distinct scaling behaviors. Gut formation is severely slowed by decreases in oxygen, while head involution and syncytial development are less impacted than the rest of development, and the order of several developmental landmarks is inverted at different oxygen levels. These data reveal that the uniform scaling seen with changes in temperature is not a trivial consequence of adjusting developmental rate. The developmental rate changes produced by changing oxygen concentrations dwarf those induced by temperature, and greatly impact survival. While extreme temperatures increase early embryo mortality, mild hypoxia increases arrest and death during mid-embryogenesis and mild hyperoxia increases survival over normoxia.

## Introduction

After discovering that the time elapsed during different morphological stages of *Drosophila* embryogenesis scale uniformly as temperature changes the overall time of embryogenesis ^1^, several colleagues questioned whether the result was surprising, suggesting instead that it was a natural and trivial consequence of physical and chemical laws. To explore this possibility, and to provide orthogonal insight into the mechanisms of the control of developmental timing, we sought to manipulate developmental rate in a temperature-independent manner.

It has long been known that oxygen levels affect the rate of animal development ^2^. In *D. melanogaster* mild hypoxia (10% oxygen) slows time to eclosion relative to normoxia (21% oxygen), while hyperoxia (41% oxygen) accelerates time to eclosion in a temperature-dependent manner. This suggested to us that studying the effects of varying oxygen levels on embryogenesis might provide an ideal complement to our earlier studies of the effects of temperature.

Although the scaling behavior of embryos grown at different oxygen concentrations has not been previously characterized, there have been extensive studies of the effect of oxygen on the *D. melanogaster* embryo. In normal development, oxygen sensation plays a crucial role in cellular differentiation, organogenesis, and growth rate. It is known to influence Notch, Wnt, and OCT4 pathways ^3^ and at low levels slows growth by driving components of the Tor pathway ^4, 5^. It is critical for hematopoiesis ^6^, myogenesis ^7, 8^, and notochord and liver formation in vertebrates ^9, 10^.

Hypoxic *Drosophila* syncytial embryos arrest at a metaphase checkpoint ^11^ and resume development under normoxia if the hypoxic period is not too long ^12^. Cellularized embryos survive longer hypoxic periods, up to several days ^13^. However, hypoxic arrest is not entirely benign, as even brief periods of hypoxia lead to smaller bodies and wings, driven in part by decreased cell size ^14– 16^. Active oxygen sensing and nitric oxide signaling drive this arrest, which is independent of the electron transport chain ^13, 17, 18^. Hypoxia tolerance also varies between tissues ^19^ and possibly between stages of embryonic development. Hyperoxia, on the other hand, is toxic ^20, 21^ and drives malformation of mitochondria ^22^.

The response of the *D. melanogaster* embryo to oxygen is highly conserved, in both function and molecular mechanism ^21, 23– 25^. *Drosophila*, like other animals, regulate metabolism and gene expression in response to changes in oxygen levels through the *HIF-1* *α* pathway, which communicates with the Tor and VEGF pathways. Under normal conditions, proline residues of *simalar* ( *sima/hif-1/HIF-1α*) are hydroxylated by prolyl hydroxylase ( *Hph/egl-9/EGLN*) to both inactivate *sima/hif-1/HIF-1α* and target it for Vhl-dependent degradation ^19, 26^. The prolyl hydroxylase *Hph/egl-9* is itself negatively regulated under hypoxia by the cystathionine *β*-synthase *Cbs/cysl-1/CBS*, an ambient oxygen sensor via hydrogen sulfide signaling. *sima* has an oxygen-dependent degradation domain with a nuclear export sequence ^27^. Thus, only during hypoxia does *sima* escape degradation and accumulate in the nucleus ^26^. Rather than serving as a switch, the process is dynamic, with greater levels of oxygen accelerating both the degradation and nuclear export of *sima* ^27^.

Over the course of development, changes in insulin levels, the metabolic state of the embryo, and temperature may impact the oxygen response ^24, 28– 31^, either directly or through its dependence on transcription, nuclear-import and export, prolyl hydroxylation and Vhl-dependent degradation ^32^.

Here we use time-lapse imaging of embryos under a range of oxygen concentrations with precise temperature control to monitor the effects on developmental timing and morphology. In covering hypoxic through hyperoxic and warm through cold conditions, we have collected dynamic data on how the developing embryo responds to oxygen, and how that response is affected by temperature.

## Methods

### Rearing and imaging of *Drosophila*

*Drosophila melanogaster*, OreR, were reared and maintained on standard fly media at 25°C. Egg-lays were performed in medium cages on 10 cm molasses plates for 1.5 hours at the temperature at which the lines were maintained after pre-clearing. Embryos were collected and dechorionated with fresh 50% bleach solution (3% hypochlorite final) for 60 seconds in preparation for imaging.

**Figure 1.**
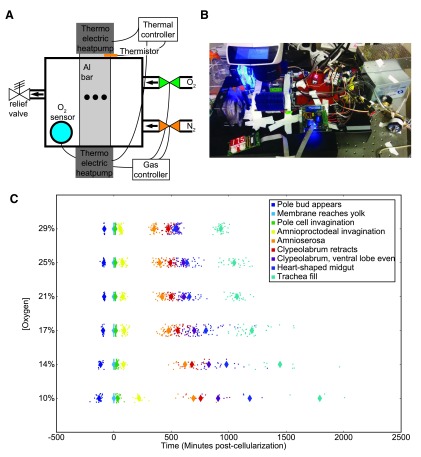
Developmental rate responds to oxygen concentrations. ( **A**) The oxygen control schematic. A thermistor embedded in the aluminum bar provides temperature data to the temperature controller, which in turn adjusts the voltage to the thermo-electric controllers (Peltier). An oxygen sensor in the airtight box provides feedback on oxygen concentrations to the gas controller, which opens and closes oxygen and nitrogen valves accordingly. Embryos are imaged in the center of the aluminum bar within the airtight box, indicated by the black dots in the schematic. ( **B**) Image of the oxygen control setup mounted on the microscope at 20% oxygen and 17.5°C. ( **C**) Developmental rate across all stages changes with the oxygen concentration, performed at 27.5°C. Each animal is represented with a dot, with averages represented with a large diamond. Developmental times here are zeroed on the end of cellularization.

Embryos were monitored by modifying a temperature control system ^1^ in which an aluminum bar was embedded in an acrylic box (TAP Plastics). Both ends of the aluminum bar were external to the box and bound to Peltier heat pumps and heat sinks. A thermistor connected to the aluminum bar provided feedback to maintain the temperature using an H-bridge temperature controller (McShane Inc., 5R7-570). Embryos were glued ^33^ to oxygen-permeable film (lumox, Greiner Bio-one), covered with Halocarbon 700 oil (Sigma), and placed over holes drilled in the aluminum for imaging. An oxygen sensor (Grove Gas sensor (O2)) was placed in the box and connected to an external computer (Arduino-style Seeeduino V3.0 (Atmega 328P)). Finally, the box was sealed with two gas inputs and an over-pressure release. The computer utilized the oxygen sensor input and controlled two valves via NPN transistors, one connected to an oxygen tank and regulator and one connected to a nitrogen tank and regulator, to maintain specific oxygen concentrations in the box ( Figure 1A).

Time-lapse imaging with bright field transmitted light was performed on a Leica M205 FA dissecting microscope with a Leica DFC310 FX camera using the Leica Advanced Imaging Software (LAS AF6000 version 2.3.5) platform. Greyscale images were saved from pre-cellularization to hatch. Z-stacked images were saved every two minutes (five minutes at 17.5°C). Analysis data available from http://dx.doi.org/10.6084/m9.figshare.1572474 and imaging data available from http://dx.doi.org/10.6084/m9.figshare.1582639.

Z-stack and image analysis were conducted as previously described ^1^. Events selected for measurement (pole-bud appearance, membrane reaching yolk, pole cell invagination, amnioproctodeal invagination, amnioserosa exposure, clypeolabrum retraction, clypeolabrum and ventral lobes being even, heart-shaped midgut, and the filling of the trachea) were identified by hand using a graphical user interface. Oxygen dependent trends were analyzed with least-squares regression. Significant differences between events in their response to oxygen changes were determined by comparing the pooled estimate of the variation about the regression line using a t-test with a Bonferonni multiple testing correction. For modeling total developmental response to oxygen and temperature changes, least-squares regression was used based on linear, exponential, logarithmic, polynomial (up to cubic), and inverse proportional models, with the models consistently yielding the best Pearson product-moment correlation coefficient being selected. For the combined effect of both oxygen and temperature, all possible combinations of exponential and inverse-proportional models identified for each component were attempted with least squares surface regression. The curve fit with the best adjust correlation coefficient $\left({\bar{R}}^{2}\right)$ across all available data was selected. All scripts are available at github.com/sgkuntz/OxygenCode.

## Results

### Oxygen concentration controls developmental rate

We used automated time-lapse imaging in an airtight box with oxygen concentration control (±1%) and precise temperature control (±0.1°C) to track development using previously described methods ^1^. We investigated embryos raised at constant oxygen concentrations (29%, 25%, 21%, 17%, 14%, and 10% *O*_2_) and kept at three different temperatures (17.5°C, 22.5°C, and 27.5°C), giving a total of eighteen specific conditions with over 800 embryos. A schematic of the setup is provided in Figure 1A. The actual setup is shown in Figure 1B.

In agreement with previous research, developmental rate correlates with oxygen concentrations ( Figure 1C). Hyperoxia accelerates development, allowing embryos to hatch sooner than they would under normal atmospheric conditions. Hypoxia slows development in a dose-dependent fashion. As oxygen levels fall, an increasing fraction of embryos die or arrest their development. Therefore, there are fewer embryos shown in Figure 1C at lower oxygen concentrations due to low rates of successful development, despite similar numbers of animals being prepared for imaging ( Table S1).

### Oxygen-dependent developmental scaling is non-uniform and temperature-dependent

**Figure 2.**
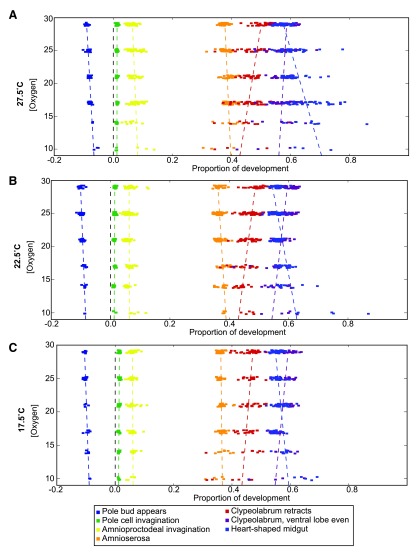
Oxygen-dependent changes vary with temperature. Gut formation and head involution are the most strikingly oxygen concentration dependent processes, but germ band retraction and syncytial development are also affected. Both syncytial development and head involution take proportionally more time as oxygen concentrations increase. Gut formation and germ band retraction, in contrast, take proportionally less time as oxygen concentrations are raised. These trends hold true across all temperatures, though the rates of change as a function of oxygen do vary. Development is normalized here between the end of cellularization and the filling of the trachea.

By tracking and analyzing nine morphological stages as oxygen concentrations change, we identified significant differences in scaling between major morphological events. While all morphological events speed up with increasing oxygen concentrations ( Figure 1C), their changes in speed are notably different. Syncytial development, as measured by the time between the appearance of the pole bud and the end of cellularization, takes proportionally less time as oxygen concentrations decrease, indicating that this stage is not slowed as much by decreasing oxygen ( Figure 2A). The stages of gastrulation (end of cellularization, pole cell invagination, and amnioproctodeal invagination) are relatively uniformly affected. Germ band retraction, as measured by amnioserosa exposure, tracks subtly but inversely with syncytial development. More striking are the oxygen-dependent changes observed in head involution (clypeolabral retraction and advancing of the ventral lobe to match the clypeolabrum) and gut formation (heart-shaped midgut). While head involution takes proportionally more time as oxygen levels increase—meaning it does not slow as much as overall development in hypoxia—gut formation does the opposite. The midgut takes proportionally less time to form as oxygen levels increase, meaning it responds more strongly to increases in oxygen than overall development. This juxtaposition of behaviors leads to an inversion of when the cephalic lobes are even versus heart-shaped midgut formation. While hypoxia leads to head involution stages finishing first, hyperoxia results in the heart-shaped midgut forming first.

**Figure S1.**
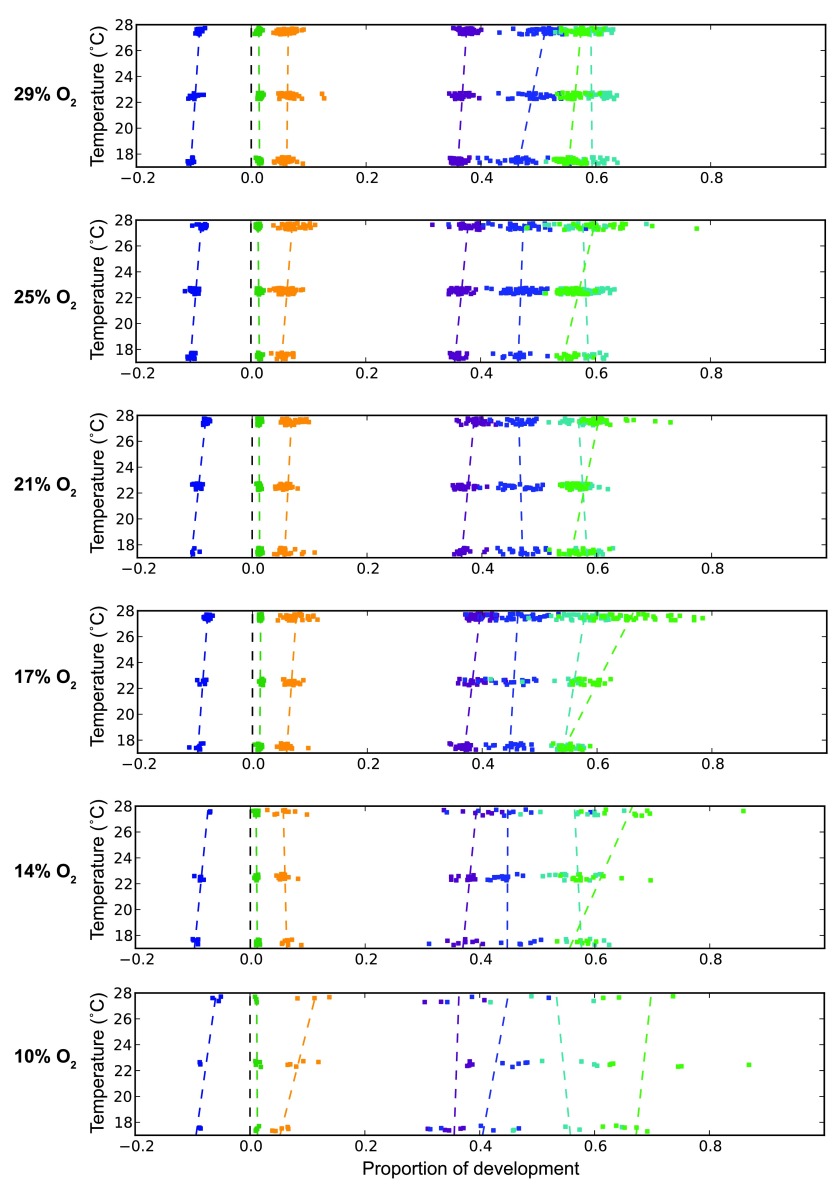
Changes in stages react differently as temperature changes. As oxygen concentrations decrease, gut-development is proportionally delayed, as can be seen with the timing of the heart-shaped midgut shifting later in development. Proportional changes in development are more severe at higher temperatures. At very low oxygen concentrations (10% oxygen), development becomes highly irregular. In many animals, development ceases during germ band retraction at very low oxygen concentrations. Development is normalized here between the end of cellularization and the filling of the trachea.

Surprisingly, the point of inversion varies with temperature ( Figure 2). At 27.5°C, the inversion takes place at 29% oxygen, while at 17.5°C the inversion falls around 19% oxygen. This may be due to an overall shift in the oxygen response curve of heart-shaped midgut formation to proportionally later in development as temperatures fall. Supplementary Figure S1 reveals how each stage of development at each oxygen concentration changes with temperature.

### Timing of death and arrest depend on both oxygen and temperature

Oxygen levels affect the stage at which embryos arrest or die. Higher concentrations of oxygen (29%) lead to more animals dying during early development, including death in the syncytium and a failure to properly gastrulate. This point of failure is similar to that observed at high temperatures with normal oxygen levels ^1^. Lethality at 25% oxygen is actually lower than that at 21%, which approximates atmospheric levels. Problems with development may be aggravated by the dechorionation and mounting procedure. At high temperatures (32.5°C) and high oxygen (29%), almost all embryos die very early in development ( Table S1).

**Figure 3.**
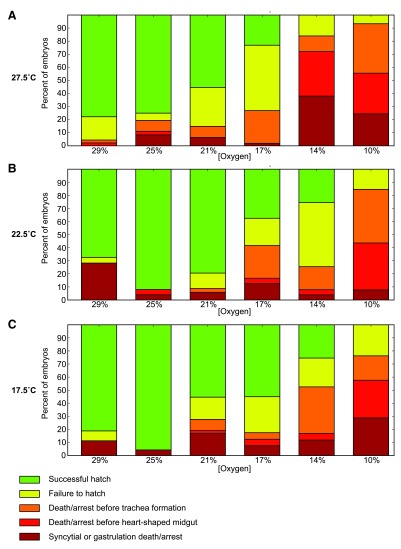
The point of failure in development depends on both oxygen and temperature. Lethality at different concentrations are shown for three different temperatures (27.5°C, 22.5°C, and 17.5°C). Lower concentrations of oxygen are more likely to exhibit failure during pre-tracheal development, with a particularly large increase in mortality between gastrulation and completion of the heart-shaped midgut (shown in red). A substantial increase in late development before trachea fill is also seen (shown in orange). Developmental arrest is frequently at germ band retraction. This is in contrast with higher oxygen concentrations, where failure is almost exclusively very early in development (shown in brick red), prior to the completion of gastrulation, or during difficulties hatching following trachea filling (shown in yellow). Highest survival is interestingly at 25% oxygen.

At lower oxygen levels there is a major shift from very early developmental arrest and death to mid-embryogenesis arrest ( Figure 3). This holds true at all temperatures (especially at 10% *O*_2_), but is most pronounced at 27.5°C, where the effects are still seen at 14% *O*_2_. Frequently development halts during germ band retraction, preventing full exposure of the amnioserosa. The midgut primordia in these embryos routinely migrates haphazardly after arrest, coinciding with the embryo falling into morphological disarray. In embryos that pass these mid-embryogenesis stages, trachea formation often proves problematic. Commonly the trachea fails to form, which coincides with arrest late in midgut formation, following the heart-shaped midgut stage. These animals generally form functional muscle, with some twitching observed.

Higher, but still hypoxic, oxygen levels (14% and 17%) have a significant fraction of embryos that fail to hatch. While embryonic development appears to be completed, including the filling of the trachea with air, larvae struggle to break out of their vitelline membrane yet fail to escape. While seen in all conditions, this behavior is most prevalent in these mildly hypoxic conditions.

### Temperature influences oxygen’s control of developmental rate

**Figure 4.**
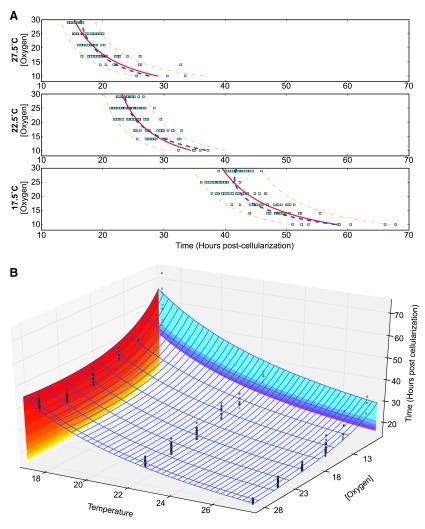
Oxygen and temperature affect developmental time cooperatively. ( **A**) Fitting at different temperatures (27.5°C, 22.5°C, 17.5°C). The shift is very temperature-dependent. The solid red line represents a fit at each particular temperature, with 90% confidence of reproduction marked with the dashed orange line. The blue dashed line represents the fit across all temperatures, which departs from the individual temperature fits. ( **B**) Total developmental time is affected by both temperature and oxygen levels. Each point represents an individual embryo at a given temperature and oxygen level. Color contours help visualize the transitions of increased heat (yellow to red contour) and increased oxygen (purple to blue contour).

**Figure S2.**
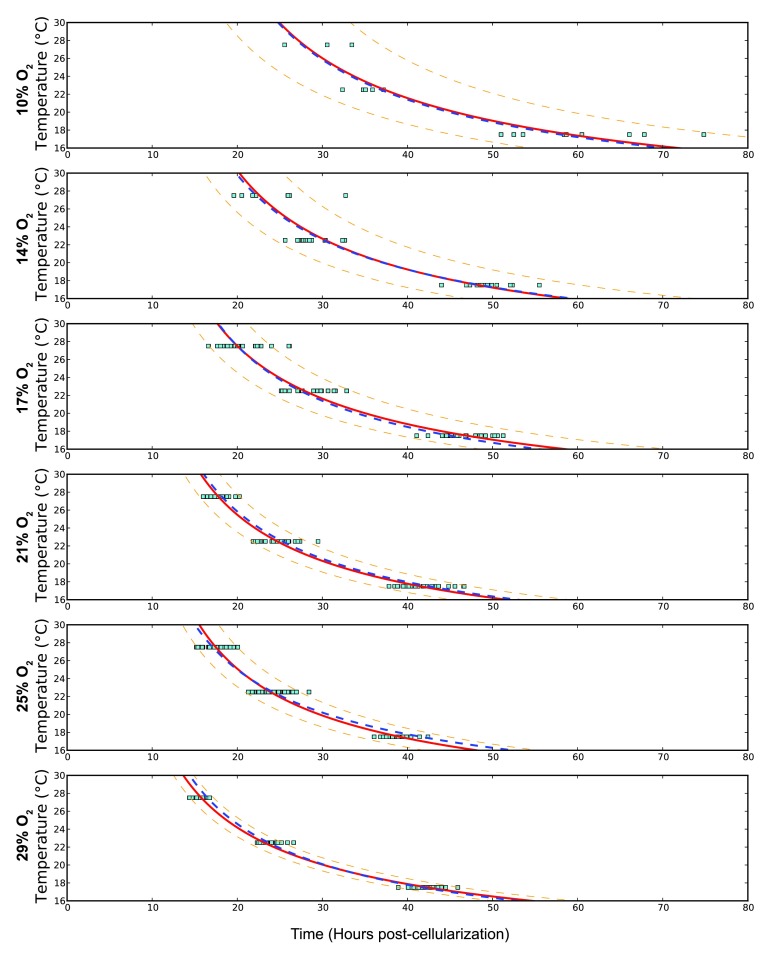
Curve fitting for each oxygen concentration as temperature changes. Curve fitting at each oxygen concentration is shown with the red line, with the 90% confidence interval delineated by the orange dashed line. The overall curve fitting is identified with the blue dashed line. Differences between different oxygen concentrations are much smaller than the range covered by temperature changes. The change in developmental time from high to low temperatures remains proportional across oxygen concentrations.

Decreasing oxygen concentrations from 29% to 10% at any temperature lead to an additional sixteen to eighteen hours of embryogenesis ( Figure 4A). This results in a different proportional change at each temperature, with nearly a 100% increase at 27.5°C and only a 50% increase at 17.5°C. This contrasts with changes in temperature, where developmental time roughly doubles over a 10°C range, regardless of the oxygen concentration ( Supplementary Figure S2).

Changes in oxygen concentration have an inverse proportional effect on developmental rate. Least squares curve fitting was attempted with multiple models, including exponential models, for changes in oxygen concentration. The data most closely matched a model based on the Monod equation. The parameters of the response for embryogenesis as a whole change significantly with temperature, however the qualitative response is the same ( Figure 4A):

$$\begin{array}{l} t_{17.5}=\frac{280.92}{\left[O_{2}\right]}+30.13\\ t_{22.5}=\frac{167.39}{\left[O_{2}\right]}+17.58\\ t_{27.5}=\frac{204.03}{\left[O_{2}\right]}+8.55 \end{array}$$

Fitting at each oxygen concentration ( Supplementary Figure S2) yields relatively good fits using an exponential Arrhenius model. These different methods of fitting can be combined and yield the best fit as a multivariable non-additive model. The overall effect of oxygen and temperature can be combined to yield ( Figure 4B):

$$t=e^{\frac{0.47[O_{2}]+31}{T}}(\frac{65.407}{\left[O_{2}\right]}+1.0)$$

Based on the fit, oxygen appears to have an effect on both the linear and exponential coefficients. This model is empirical and does not predict effective oxygen concentration as a function of temperature-dependent changes in oxygen solubility and diffusion. Increased oxygen may allow some additional growth acceleration, but the acceleration of growth rate appears to be leveling off, asymptotically approaching a maximum. At lower oxygen levels, the prevalence of arrest is expected to overtake the observed response curve.

## Discussion

We tracked embryogenesis at different oxygen concentrations to determine its effect on development, performing these experiments in conjunction with precise temperature control. We found that developmental rate is highly dependent on oxygen and exhibits a complex relationship with temperature. Embryos are not as robust to oxygen changes and have much less of a dynamic response than is seen with temperature. We observed significant differences in oxygen responsiveness across tissues and morphological events. These changes can be aggravated by temperature (long known to interact with oxygen consumption ^24, 34^) to reveal situations in which embryogenesis loses its uniform thermal scaling.

The prevalence and timing of developmental failure depend strongly on oxygen. Under hypoxia, failure is largely concentrated in mid-embryogenesis at germ band retraction. Commonly the germ band fails to fully retract to expose the amnioserosa. It is possible that this stage either requires more oxygen or its complexity makes it prone to failure. A checkpoint at this stage that hypoxic embryos fail to pass may explain this phenotype. Rapid hypoxic arrests are not frequently observed under our conditions. Oxygen concentrations of 10% and 14% may fail to trigger complete hypoxic arrest in a subset of embryos yet serve to slow development enough to cause problems. Increasing oxygen levels would likely restart development, but the manner in which it restarts would depend on the stage of arrest, duration of hypoxia, and revived oxygen levels. Thermal tolerance has been previously linked to oxygen concentration ^34– 36^. Likewise, we see increased hypoxic mortality with increased temperature.

Hypoxia’s mid to late embryogenesis failure contrasts with high heat and high oxygen, where failure occurs during early development, during either the syncytium or early gastrulation. Under conditions with high oxygen tension, death frequently resembles, at least qualitatively, high temperature normoxia death. Failure during syncytial development commonly involves mass migration of nuclei throughout the embryo, making it difficult to distinguish the point of failure between pre-gastrulation death resulting in nuclei migration and premature gastrulation that causes death.

The syncytium responds differently to oxygen levels than other embryonic stages. Perfect scaling collapses in the syncytium at high temperatures ^1^, so it is not surprisingly that a difference is seen with the oxygen response as well. Interestingly, while syncytial development is less responsive to changes in oxygen than other stages across the range we tested, it is more responsive to excess heat than other stages. The difference may be aggravated by the lack of transcriptional responses available at that stage and the limited repertoire of maternally deposited genes and mRNAs. This may lead to the syncytium lacking high heat mitigations and prophylactic hypoxic responses. This implies that transcriptionally active embryos deliberately slow development either under high heat when kinetics are accelerating or under hypoxia to conserve energy.

The developmental rate response to oxygen is more subtle, yet causes more problems, across the range we tested than is seen with a moderate change in temperature. We measured ambient oxygen, meaning the exact oxygen concentration in the embryo microenvironment may differ. Across a 10°C differential, developmental time doubles with minimal change in viability. This is virtually invariant, regardless of oxygen concentrations. However, across a 19% change in oxygen concentration, development time experiences an absolute, rather than proportional, change of sixteen to eighteen hours. Changes in oxygen thus provide a proportionally smaller change in developmental time with enormous consequences for viability. While changes in temperature follow the Arrhenius equation, changes with oxygen appear to follow Monod’s equation. Rather than a logarithmic curve, developmental time is inversely proportional to oxygen concentration. This comparatively shallow oxygen response undermines the hypothesis, which had previously been refuted for thermal limits ^37, 38^, that oxygen availability explains temperature-dependent changes. Changes in temperature will affect oxygen diffusion in the embryo, with a 10°C change shifting the effective oxygen concentration by ∼4%. However, the difference in developmental time between 21% and 17% oxygen at 27.5°C is dwarfed by the dramatically larger difference between 27.5°C and 17.5°C at 21%. Therefore, basic energy metabolism is not solely responsible for the changes in developmental rates seen across temperature. Our results show that the embryo’s developmental program is robust to small changes in oxygen. This suggests some leeway in respiration efficiency; nevertheless, there is a notable biological response.

## Data availability

*Figshare*: Raw data for Kuntz and Eisen, 2015 ‘Oxygen changes drive non-uniform scaling in *Drosophila melanogaster* embryogenesis’. 10.6084/m9.figshare.1582639 ^39^

*Figshare*: Oxygen imaging. 10.6084/m9.figshare.1572474 ^40^

## Figures and Tables

**Table S1.** Point of Failure.

Oxygen Concentration	Temperature	Syncytial or Gastrulation Death		Death before Heart-shaped Midgut		Death before Trachea Formation		Failure to Hatch		Successful Hatch		Total
29%	32.5°C	26	87%	4	13%	-	-	-	-	-	-	30
	27.5°C	-	-	1	2%	1	2%	8	18%	35	78%	45
	22.5°C	13	28%	-	-	-	-	2	4%	31	67%	46
	17.5°C	6	11%	-	-	-	-	4	8%	43	81%	53
25%	27.5°C	3	8%	1	3%	3	8%	2	6%	27	75%	36
	22.5°C	2	4%	2	4%	-	-	-	-	46	92%	50
	17.5°C	1	4%	-	-	-	-	-	-	22	96%	23
21%	27.5°C	3	6%	-	-	4	9%	14	30%	26	55%	47
	22.5°C	2	6%	-	-	1	3%	4	12%	27	79%	34
	17.5°C	8	17%	1	2%	4	9%	8	17%	26	55%	47
17%	30.0°C	40	98%	1	2%	-	-	-	-	-	-	41
	27.5°C	1	2%	-	-	13	25%	26	50%	12	23%	52
	22.5°C	6	13%	2	4%	12	25%	10	21%	18	38%	48
	17.5°C	3	8%	2	5%	2	5%	11	28%	22	55%	40
14%	27.5°C	19	38%	17	34%	6	12%	8	16%	-	-	50
	22.5°C	2	4%	2	4%	9	18%	25	49%	13	25%	51
	17.5°C	7	12%	3	5%	21	36%	13	22%	15	25%	59
10%	27.5°C	11	24%	14	31%	17	38%	3	7%	-	-	45
	22.5°C	3	8%	14	36%	16	41%	6	15%	-	-	39
	17.5°C	17	29%	17	29%	11	19%	14	24%	-	-	59
