# Supplementary material for: Oxygen changes drive non-uniform scaling in Drosophila melanogaster embryogenesis
Source: F1000Res. 2015 Oct 23;4:1102. [Version 1] doi: 10.12688/f1000research.7221.1 (PMC4670008; doi:10.12688/f1000research.7221.1)
Supplement: Peer review history

# Peer review: Referee response for version 1

**DOI:** 10.5256/f1000research.7779.r11301

**Published:** 2015-11-23

**Authors:** Pablo Wappner

**Affiliations:** 1 Leloir Institute Foundation, Buenos Aires, Argentina

Kuntz and Eisen have analysed the effect that variations of oxygen concentrations and temperature exert on *Drosophila* embryonic development. A previous study from the same group had shown that temperature variations affect embryogenesis preserving the order and relative duration of a list of morphological events during this developmental process. The question addressed in this work is as whether variations of oxygen levels also preserve the order and relative duration of events during embryogenesis. They have designed and built a special device to monitor embryogenesis while controlling oxygen levels and temperature simultaneously. The work is technically sound and the results are truly unexpected: The different morphological events of the embryogenesis are not affected by oxygen levels to a similar extent, but rather, each morphological event responds to such variations in an unequal manner. Strikingly, the order in which some of the morphological events occur is altered when oxygen levels are modified. These effects are modulated by the temperature, so that the order and relative duration of each of the events of the embryogenesis is independently modulated by oxygen and temperature simultaneously. The approach and the whole concept is highly original and adds a new dimension to our understanding of metazoan development that paves the road for further studies aimed to the definition of the molecular and genetic mechanisms that underlie this phenomenon.

I have read this submission. I believe that I have an appropriate level of expertise to confirm that it is of an acceptable scientific standard.

# Peer review: Referee response for version 1

**DOI:** 10.5256/f1000research.7779.r11218

**Published:** 2015-11-16

**Authors:** Angela H. DePace

**Affiliations:** 1 Department of Systems Biology, Harvard Medical School, Boston, MA, USA

This study assesses the effect of oxygen level and temperature on developmental timing in Drosophila embryos. The methods are adequately described to interpret the experiments. Oxygen and temperature are controlled externally in a custom built apparatus. Developmental timing is assessed by time lapse imaging under bright field; developmental milestones were manually annotated from this data. The statistical analysis used to compare across conditions is adequately described.  This experimental set up does not make any internal measurements of oxygen level or temperature, which may be of interest for future studies, but this is a substantial technical undertaking.

The conclusions are primarily descriptive—the relative duration and timing of developmental stages varies in a non-uniform way with oxygen level and the effect depends on temperature.  This result contrasts with the effect of varying temperature alone, where developmental stages maintain precise relative timing despite variation of the overall time of development, as described in a previous study by the same authors. The authors also pinpoint which stages and tissues are most sensitive to changes in oxygen level. These phenomenological conclusions will be useful for future studies.

There are also some secondary interpretations based on their phenomenological observations. 

First, because the blastoderm stage is more responsive to changes in temperature than other stages, but less responsive to changes in oxygen, the authors speculate that this is because blastoderm embryos have a limited ability to mitigate heat stress via transcription of new genes or translation of existing mRNA. Reciprocally, they state that “transcriptionally active embryos” (I read this to mean non-blastoderm embryos) “deliberately slow development either under high heat when kinetics are accelerating or under hypoxia to conserve energy.”  In my opinion, this interpretation doesn’t immediately follow from the previous observation about the heat and oxygen sensitivity of blastoderm embryos.  It is also not clear what the authors mean by “deliberately”; perhaps they mean that the embryos have pathways dedicated to a regulated response to these conditions, rather than the effect arising passively from global biochemical parameters.

Second, the authors state that the thermal response is not due to changes in oxygen delivery as the two responses have distinct dynamics and phenotypic outcomes.  This generalizes previous results about oxygen availability under extreme temperatures, as pointed out by the authors.  In my view, this interpretation is sound.

In my opinion, one of the most interesting directions for this line of research is how variation in temperature and oxygen level are sensed and how responses are executed.  In the previous paper, the authors stated that they expected non-uniform scaling in response to temperature, because chemical reactions are known to exhibit different temperature sensitivity. Under this line of thinking, pathways specific to distinct developmental stages would respond differently to temperature, resulting in heterochrony.  Instead, they do find uniform scaling of developmental timing in response to temperature. In this work, specific pathways are known to have differential sensitivity to oxygen (as described in the introduction), and indeed they observe heterochrony.  What underlies this difference?  Are developmental pathways not differentially sensitive to temperature (or stated reciprocally, do they all respond to temperature the same way)?  Or are different molecular or pathway level effects of temperature buffered by other systems? Both of these are interesting possibilities.  Deciphering whether uniform temperature sensitivity and/or buffering are under selection will also be exciting.

I have read this submission. I believe that I have an appropriate level of expertise to confirm that it is of an acceptable scientific standard.

# Peer review: Referee response for version 1

**DOI:** 10.5256/f1000research.7779.r10904

**Published:** 2015-11-10

**Authors:** Sofia Lavista Llanos

**Affiliations:** 1 Department of Evolutionary Neuroethology, Max Planck Institute for Chemical Ecology, Jena, Germany

**Motivation:**

Authors have previously shown for various *Drosophila* species, that the relative timing of events within embryogenesis is temperature indifferent (ref 1 in manuscript). To test if this uniform scaling with varying temperature is an intrinsic property of developing embryos, or rather a specific response to thermal fluctuations, authors soughed a temperature-independent manner to manipulate the embryogenesis development rate.

**Proposed study:**

Authors studied the development scale of embryos grown at different oxygen concentrations and its interaction with varying temperatures.

**Main result found:**

In contrast to the uniform scaling of development with temperature variation, authors found heterochrony of the developmental events with changes in oxygen levels. There was an interaction between temperature and oxygen.

**Conclusions:**

*These data reveal that the uniform scaling seen with changes in temperature is not a trivial consequence of adjusting developmental rate.*

Authors inaccurately raise conclusions on the behaviour of developing embryos with fluctuating temperature (referred as to the previous study), based on experiments performed with varying oxygen in the present study *.*

Instead, the data presented in this study supports the hypothesis that a uniform scaling of development is not an intrinsic property of developing embryos. Rather, embryos adjust their developmental program in different ways depending on variable ambient conditions.

**Relevance of the study:**

Considering the importance of studies on tissue and cellular responses to hypoxia, for human health (i.e.: tumour growth and cardiovascular disease, among others) this study could potentially become of great impact.

**Control aspects that should be addressed:**

1. Authors assume that O _2_ is delivered uniformly through the embryo, as most probably temperature does. Are the results here obtained simply a consequence of a differential diffusion of O _2_ through the embryonic tissues?
2. Does temperature affect O _2_ diffusion, explaining the interaction between both variables here shown?
3. Is there an effect of nitrogen changing concentration in the hypoxia treatments?

**Derived biological questions & suggestions:**

1. Is the oxygen dependency of developmental rate conserved in the different *Drosophila* species?
2. The non-uniform scaling of development in hypoxia might indicate that oxygen plays a signalling role during the developmental process (see Jarecki *et al., *1999).
3. Might mild physiological hypoxia in embryonic deep tissues explain the differential reluctance to oxygen deprivation of different tissues?
4. Is the embryonic developmental program a coordinated hierarchical chain of events, being the chronological appearance of each one dependent on the occurrence of the previous event? Or, rather, is the embryonic developmental program a sum of independent events each following an autonomous synchrony and/or independent scaling that adjusts to the ambient conditions? Or a combination of both? These aspects could actually be addressed in the manuscript based on the results obtained with varying temperature (previous study) and varying oxygen (results shown here).
5. Authors could put their results into an ecological context, comparing species with different habitats and ovipositon sites.
6. These studies were done on de-chorionated embryos. Authors could address the fact that chorion might serve as a physiological barrier to fluctuating oxygen concentrations, which would prevent desynchronising the embryonic development with varying ambient conditions.

**Proposed experiments:**

1. To reinforce the evidence of the role of oxygen/hypoxia on the development scale, authors could use mutants of the oxygen cellular sensing machinery (i.e.: Hph (CG31543), sima (CG7951); see Centanin *et al., *2005) in time-lapse imaging experiments. Such experiments could serve to rule out a mere physic-chemical effect of hypoxia on the embryonic development scale and **at the same time** test if oxygen has an active signalling role during embryogenesis.
2. Authors could follow with fluorescent markers the dividing cells (e.g.: bromouridine) and or apoptotic cells (e.g.: TUNEL) during development at varying oxygen levels to get an insight on the cellular/molecular mechanisms sustaining the adjustment of the development to varying oxygen or temperature fluctuations.
3. Authors could try to address the embryonic internal oxygen concentrations by indirect labelling of populations of synchronized embryos (30 min egg-laying) fixed after the relevant oxygen/temperature treatments (e.g.: lac-Z/GFP staining using hypoxia reporter genes, NADPH diaphorase staining).
4. To test if the presence of chorion affects the response to varying oxygen, authors could repeat the experiments with synchronized chorioned eggs (30 min egg-laying) & immediately dechorionated + fixed after treatment. This would test if indeed the ambient variation in oxygen affects development in nature.
5. Having overcome the challenge of obtaining developmental staged-matched samples for different species in their previous study (ref 1 in manuscript), authors could include different species to complement this study.

 

**Minor comments:**

1. **Introduction paragraph-5 line-6:** *simalar* should read ‘similar’.
2. **Methods:** *Egg-lays were performed in medium cages on 10 cm molasses plates for 1.5 hours at the temperature at which the lines were maintained after pre-clearing. *This phrase is not clear. Were adult flies maintained at the same temperature as eggs? Does this affect the stage at which eggs are laid? With 1.5 hours collection eggs have an error of ± 45 minutes, which at the beginning of embryogenesis might have huge effects on development.
3. How was the proportion of development calculated? Data quantification methods should be clearly detailed in methods section.
4. **Figure 1:** Y axis is not in scale. Authors should also indicate in the figure legend the number of eggs ( *n*) for each experiment.
5. **Results paragraph-3 line-5:** *Syncytial development, as measured by the time between the appearance of the pole bud and the **end of cellularization**, takes proportionally less time as oxygen concentrations decrease, **indicating** that this stage is not slowed as much by decreasing oxygen.*
6. The event *‘end of cellularization’ *is not shown on figure 1C legend.
7. Authors might want to re-phrase the passive voice in *‘indicating that this stage is not slowed as much by decreasing oxygen’ *for ‘ indicating that decreasing oxygen does not slow this stage’.
8. I don’t see the dependence of one argument with the other. Authors might want to rephrase the entire argument.
9. **Results paragraph-3:** The events mentioned in the text do not correspond to those shown in the figure legend. Authors might want to make it easier for the reader to follow their descriptions by choosing a uniform way of representing the data and referring to it in the text.
10. **Figure 2:** Legend reference does not correspond to the events described in the figure legend, making difficult for the reader to follow the arguments.
11. **Table S1:** legend missing; it might aid clarification to provide a legend to this table.
12. **Figure S1:** Title is not clear: Changes in stages react differently as *temperature changes. *What do they react to?
13. **Figure S1: **No reference legend is provided!
14. **Discussion pagraph-2 line-6:** *A checkpoint at this stage that hypoxic embryos fail to pass may explain this phenotype*. Phrase not clear.
15. **Discussion: pagraph-5 line-3:** Authors might suggest experiments to grasp this aspect.
16. **Discussion:** final conclusion does not correspond to the aim stated at the beginning of the manuscript.

**Final comment: ** This study addresses an important topic relevant to biological and medical fields. Despite the fact that the conclusions raised are rather superficial, and regrettably inadequate to the objective aimed in the first place **after major revision (!)**, this manuscript could make an important contribution to *Drosophila* embryogenesis and, more general, to homeostasis of animal development.

I have read this submission. I believe that I have an appropriate level of expertise to confirm that it is of an acceptable scientific standard, however I have significant reservations, as outlined above.
